# Exploring the Potential Roles of *SLC39A8* and *POC5* Missense Variants in the Association Between Body Composition, Beverage Consumption, and Chronic Lung Diseases: A Two-Sample Mendelian Randomization Study

**DOI:** 10.3390/ijms26167799

**Published:** 2025-08-12

**Authors:** Oladayo E. Apalowo, Hunter K. Walt, Tolu E. Alaba, Joel J. Komakech, Mark W. Schilling

**Affiliations:** 1 Department of Biochemistry, Nutrition and Health Promotion, Mississippi State University, Starkville, MS 39762, USA; hkw59@msstate.edu (H.K.W.); koma2jr@gmail.com (J.J.K.); mws72@msstate.edu (M.W.S.); 2 F. Widjaja Foundation Inflammatory Bowel Disease Institute, Cedars-Sinai Medical Center, Los Angeles, CA 90048, USA; tolualaba1@gmail.com; 3 Department of Family and Consumer Sciences, North Carolina A&T State University, Greensboro, NC 27411, USA

**Keywords:** body composition, obesity, asthma, COPD, biomarker, Mendelian randomization

## Abstract

The study examined the association between body composition and beverage consumption and the risk of asthma and chronic obstructive pulmonary disease (COPD) and explored the single nucleotide polymorphisms (SNPs) involved in these associations by leveraging summary statistics from genome-wide association studies (GWAS) in nonoverlapping populations. The IEU OpenGWAS project was sourced for exposure datasets: body mass index, body fat percentage, fat-free mass, total body water mass, alcohol intake frequency, and coffee intake, and selected health outcome datasets: asthma and chronic obstructive pulmonary disease. Datasets were assessed and filtered using R, followed by a two-sample Mendelian randomization analysis. The MR Egger, weighted median, inverse variance weighted, simple mode, and weighted mode methods were used to examine the association between exposures and outcomes. Heterogeneity and pleiotropy analyses were used to evaluate the reliability of results. Additionally, SNPnexus was used to ascertain SNPs linked to established phenotypes, while SNP annotation was obtained from the Ensembl BioMart database via the biomaRt package. Genes belonging to overlapping groups were visualized using ComplexHeatmap. Higher body fat percentage (OR = 1.72, 95% CI: 1.23–2.41, *p* = 0.002), increased BMI (OR = 1.56, CI: 1.23–1.20, *p* = 2.53 × 10^−4^), and more frequent alcohol intake (OR = 1.34, CI: 1.08–1.68, *p* = 0.009) were associated with elevated COPD risk. Asthma risk was similarly increased with higher body fat percentage (OR = 1.60, CI: 1.23–2.21, *p* = 0.001), BMI (OR = 1.54, CI: 1.29–1.84, *p* = 2.23 × 10^−6^), fat-free mass (OR = 1.21, CI: 1.02–1.44, *p* = 0.032), and alcohol intake frequency (OR = 1.19, CI: 1.01–1.40, *p* = 0.039). Total body water mass and coffee intake were not associated with asthma and COPD. SNP annotation revealed that some genetic variants that influenced the association of the exposure variables with asthma and COPD were missense variants in several genes, including the evolutionarily highly conserved gene, *SLC39A8* (rs13107325; C/A/T allele), and *POC5* (rs2307111; T/A/C allele), as well as intronic variants in *FTO* (rs56094641; A/G/T allele) and *NRXN3* (rs10146997; A/G allele). The discovery of the missense variants rs13107325 and rs2307111 in *SLC39A8* and *POC5*, respectively, in addition to other intronic and synonymous SNPs suggests that these SNPs may have some roles in the development or progression of asthma and COPD. This may contribute to the identification of molecular signatures or biomarkers that forecast the risk, development, or therapeutic response of chronic lung diseases in persons with metabolic dysregulation, including obesity.

## 1. Introduction

Chronic respiratory diseases (CRDs) affected an estimated 545 million people and contributed to 3.91 million fatalities worldwide in 2017 [1,2]. Chronic obstructive pulmonary disease (COPD) and asthma are the predominant types of CRDs linked to demographic trends, socioeconomic landscape, and risk factors such as tobacco use, environmental and occupational pollutants, and metabolic issues [1]. COPD is a progressive condition of airflow obstruction with the presence of inflammation, which can affect other organ systems. COPD is the fifth leading cause of mortality and is projected to rise to third by 2030 [3]. As populations age, the burden of COPD-related hospitalizations continues to grow [4]. Cigarette smoking remains the primary risk factor, though environmental pollutants also contribute [5]. Beyond COPD, smoking increases the risk of other respiratory conditions, including asthma [1], a heterogeneous inflammatory airway disease which is associated with allergic reactions, characterized by distinct phenotypes, principally differentiated by characteristics such as age of onset, smoking history, exacerbation frequency, underlying genetics, and obesity prevalence [6]. Common symptoms such as wheezing, cough, and shortness of breath are managed primarily through inhaled therapies, though comorbidities can exacerbate severity in difficult-to-treat cases [3].

Parental smoking has been linked to increased incidence of childhood asthma and wheeze [7,8]. A 16-year prospective cohort study that examined active and passive smoking behavior among US black women reported a significant relationship between smoking and the risk of adult-onset asthma [9]. There has been a substantial rise in the elderly population over the last three decades [10]. During this period, smoking decreased by 28.4% in males and 34.4% in females, with notable regional variation [5]. Furthermore, sociodemographic landscape, economic circumstances, and risk exposure patterns have changed, hence modifying the trajectory of CRDs [1]. Besides smoking, another major risk factor for CRDs, particularly asthma, is obesity [11]. Asthma incidence, respiratory symptom prevalence, deteriorating lung function, insufficient disease management, and reduced effectiveness of asthma treatment are all linked to a high body mass index (BMI ≥ 25 kg/m^2^) [12,13,14,15]. Furthermore, additional epidemiological data indicated that obesity has become increasingly common among people with COPD [16,17]. The increasing interest in elucidating the molecular pathways associated with obesity-related outcomes in both COPD and asthma suggests that body composition prior to and throughout the course of the disease may be a critical factor that contributes to the onset and progression of both conditions. Moreover, dietary factors, including alcohol consumption, have been identified as a trigger for asthma [18,19].

According to the World Health Organization (WHO), the global obesity rate has doubled over the last three decades. In 2022, 16% of 18-year-olds were obese worldwide, and over 390 million 5–19-year-olds were overweight. Overweight (including obesity) among 5–19-year-olds rose from 8% in 1990 to 20% in 2022 [20]. Once considered an issue of high-income nations, the rates of overweight are rising in low- and middle-income countries, with approximately 12.1% more African children under five classified as overweight since 2000. In 2024, more than half of the children under the age of five who were classified as obese or overweight resided in Asia [20]. Obesity is defined as a BMI of ~30 kg/m^2^. Traditional nutritional evaluation, which uses BMI to diagnose overweight and obesity, can misclassify. The relationship between BMI and body fat percentage is nonlinear and differs between genders [21]. Hence, the Lancet Commission on Clinical Obesity suggests adding waist circumference and direct fat measurement to BMI to reduce obesity misdiagnosis [22].

Genetic epidemiology studies the heritable aspects of disease risk and susceptibility and may lead to a more robust understanding of environmental determinants of disease such as dietary factors, occupational exposures and health related behaviors that are relevant to the whole population and not only genetically susceptible subpopulations [23]. This research method, known as Mendelian randomization (MR), employs genetic variants discovered in genome-wide association studies (GWAS) as instrumental variables (IVs) and has been used extensively to infer associations between exposures and outcomes using observational data [24,25,26,27]. The MR analysis is based on Mendel’s second law, which states that parental alleles are randomly assigned to children during gamete production, regardless of environmental or socioeconomic circumstances [27]. Because this strategy mimics a randomized controlled trial (RCT), causal interpretations are more likely, especially when it uses genetic variants to eliminate bias related to confounding and reverse causation [23]. The genetic variants must fulfill the criteria of an IV: strong association with the exposure of interest, association with the outcome only via the exposure (indicating no horizontal pleiotropy), and lack of association with any confounder [28,29].

The identification of thousands of disease-relevant variants has been made possible by the systematic characterization of large human cohorts for a specific trait of interest. Population biobanks enable researchers to simultaneously investigate numerous traits and diseases and establish correlations between previously unrelated phenotypes [30]. The current study employed a two-sample MR method to examine the associations between body composition and beverage consumption and the risk of asthma and COPD. Recognizing that SNPs identified in GWAS and MR studies may not directly correspond to biologically functional variants, SNP annotation was utilized to explore the genetic variants underlying these associations.

## 2. Results

### 2.1. Genetic Variants (IVs) Selection

The strength of association was analyzed between six distinct exposures and CRDs (Table 1). The number of participants included in the exposure ranged from 428,860 to 462,346 and they were of European descent. The outcomes comprised 17,438 cases and 131,051 controls for asthma, along with 657 cases and 210,300 controls for COPD. The number of SNPs used varied from 36 to 99 (Table 2), with all F-values exceeding 10, which indicates a low risk of weak bias and the reliability of our findings.

### 2.2. Association Between Body Fat Percentage and COPD and Asthma

Body fat percentage had a strong association with the risk of COPD (OR = 1.72, 95% CI: 1.23–2.41, *p* = 0.002) and asthma (OR = 1.60, 95% CI: 1.23–2.21, *p* = 0.001) using the IVW method (Table 2). The weighted median (OR = 1.72, 95% CI: 1.23–2.41, *p* = 0.002) and weighted mode (OR = 2.32, 95% CI: 1.25–4.29, *p* = 0.001) methods also confirmed similar results for asthma. Table 3 shows no heterogeneity among the SNPs for COPD, as the *p*-value for Cochran’s Q statistic exceeded 0.05. In contrast, for asthma, the Cochran’s Q statistic revealed heterogeneity among the IVs (*p* = 0.017), potentially due to data from various consortiums, yet this does not influence the main conclusions of the analysis. Moreover, the MR Egger regression intercept was applied to assess the robustness against horizontal pleiotropy. Our results indicated a lack of horizontal pleiotropy, indicating a lack of effect on its association with COPD and asthma (*p* > 0.05, Table 3). The funnel plot showed no signs of asymmetry, and the leave-one-out analysis did not uncover any individual SNPs that significantly altered the results (Supplementary Figure S1). During gene annotation of the SNPs that were associated with the effect of body fat percentage on asthma and COPD, five genes (myotubularin related protein 11 (*MTMR11*), solute carrier family 39 member 8 (*SLC39A8*), Protein of centriole 5 (*POC5*), WSC domain-containing protein 2 (*WSCD2*), and Src homology 2 (SH2) domain-containing transforming protein B adaptor protein 1 (*SH2B1*)) were identified that harbored missense variants, along with a 3-prime untranslated region (3’UTR) variant in Fas apoptotic inhibitory molecule 2 (*FAIM2*) and several genes with intronic variants (Supplementary Files S1 and S2).

### 2.3. Association Between BMI and COPD and Asthma

BMI had a strong association with the risk of COPD (OR = 1.56, 95% CI: 1.23–1.98, *p* = 2.53 × 10^−4^) and asthma (OR = 1.54, 95% CI: 1.29–1.84, *p* = 2.23 × 10^−6^) through the IVW method (Table 2). Moreover, various approaches, including the weighted median (OR = 1.68, 95% CI: 1.35–2.10, *p* = 3.99 × 10^−6^), simple mode (OR = 1.83, 95% CI: 1.07–3.11, *p* = 0.033), and weighted mode (OR = 1.65, 95% CI: 1.23–2.19, *p* = 0.002) methods yielded similar conclusions for asthma. For COPD, Cochran’s Q statistic (*p*= 0.178) indicates no heterogeneity among SNPs. In contrast, for asthma, the Cochran’s Q statistic indicated heterogeneity among the IVs (*p* = 0.005). Moreover, the MR Egger regression intercept suggested no directional pleiotropy for both COPD (*p* = 0.334) and asthma (*p* = 0.919) (Table 3). Furthermore, the funnel plot displays no signs of asymmetrical departure, and the leave-one-out analysis did not reveal any specific SNP that influenced the outcomes (Supplementary Figure S2). Missense variants associated with the effect of BMI on asthma and COPD were identified in *SLC39A8*, *POC5*, anaphase promoting complex subunit 4 (*ANAPC4*), and *SH2B1*, along with a 3’UTR variant in *FAIM2* (only COPD) and adenylate cyclase 9 (*ADCY9*), a 5’UTR variant in G protein-coupled receptor 61 (*GPR61*), a synonymous variant in TAO kinase 2 (*TAOK2*), and several genes with intronic variants (Supplementary Files S3 and S4).

### 2.4. Association Between Fat-Free Mass and COPD and Asthma

Fat-free mass did not have a significant association with the risk of COPD (OR = 1.27, 95% CI: 0.98–1.64, *p* = 0.073). Furthermore, no association was detected using the other four methods (Table 2). However, a strong association was detected with asthma (OR = 1.21, 95% CI: 1.02–1.44, *p* = 0.032) using the IVW methods, and these findings were further supported by complementary approaches: MR Egger (OR = 1.81, 95% CI: 1.10–2.98, *p* = 0.023) and weighted mode (OR = 1.84, 95% CI: 1.05–3.23, *p* = 0.037) (Table 2). Although Cochran’s Q statistic indicated significant heterogeneity among the IVs (*p* < 0.05) concerning the association between fat-free mass and asthma and COPD, the MR Egger intercept indicated no significant directional pleiotropy (asthma; *p* = 0.097, COPD; *p* = 0.804) (Table 3). The funnel plot shows no asymmetrical departure, and the leave-one-out analysis indicated that no SNP influenced the results (Supplementary Figure S3). Gene annotation of SNPs associated with the effect of fat-free mass on asthma identified missense variants in ectonucleotide pyrophosphatase/phosphodiesterase 2 (*ENPP2*), nuclear receptor corepressor 2 (*NCOR2*), apoptosis associated tyrosine kinase (*AATK*), and *POC5*; 3’UTR variants in zinc finger CCCH-type containing 4 (*ZC3H4*), small nuclear ribonucleoprotein 13 (*SNU13*), *FAIM2*, tumor protein p53 (*TP53*), and lysine demethylase 2A (*KDM2A*); and intronic variants in other genes (Supplementary File S5).

### 2.5. Association Between Total Body Water Mass and COPD and Asthma

Total body water mass was not significantly associated with COPD (OR = 1.27, 95% CI: 0.98–1.65, *p* = 0.071) and asthma (OR = 1.18, 95% CI: 0.99–1.40, *p* = 0.068) through the IVW method (Table 2). However, additional complementary approaches such as MR Egger (OR = 2.04, 95% CI: 1.24–3.34, *p* = 0.006) and weighted mode (OR = 1.77, 95% CI: 1.03–3.04, *p* = 0.043) (Table 2) revealed that body water mass was associated with the risk of asthma. Cochran’s Q statistic indicated significant heterogeneity among the IVs (*p* < 0.05) in the association of body water mass with asthma and COPD. However, while the consequences of the MR Egger intercept revealed no directional pleiotropy for COPD (*p* = 0.799), the MR Egger intercept test for asthma indicated significant pleiotropy (*p* = 0.022) (Table 3). Further analysis with MR-PRESSO did not identify any significant outlier variants, which indicates that pleiotropy is likely present but small across the IVs, and that there is no evidence that results are due to problematic variants. Scatter, funnel, and leave-one-out sensitivity plots are included in Supplementary Figure S4.

### 2.6. Association Between Alcohol Intake Frequency and COPD and Asthma

Alcohol intake frequency had a strong association with the risk of COPD (OR = 1.34, 95% CI: 1.08–1.68, *p* = 0.009) and asthma (OR = 1.19, 95% CI: 1.01–1.10, *p* = 0.039) through the IVW method (Table 2). Additional complementary analysis with the weighted median method (OR = 1.25, 95% CI: 1.01–1.54, *p* = 0.040) yielded similar conclusions for asthma. Cochran’s Q statistics revealed heterogeneity between the SNPs (*p* < 0.05) for both asthma and COPD but no directional pleiotropy (*p* > 0.05) according to the MR Egger intercept test (Table 3). There are no indications of asymmetrical departure in the funnel plot, and the leave-one-out analysis did not identify a particular SNP that clearly affected the results (Supplementary Figure S5). Missense variants in alcohol dehydrogenase 1B (Class I) (*ADH1B*), protein phosphatase 2 regulatory subunit B (*PPP2R2D*), and *ANAPC4*; 3’UTR variants in member RAS oncogene family (*RAB30*), argonaute RISC catalytic component 2 (*AGO2*), Huntingtin (*HTT*), electron transfer flavoprotein alpha subunit (*ETFA*), and nuclear fragile X mental retardation-interacting protein 2 (*NUFIP2*); and many genes with intronic variants were associated with the effect of alcohol intake frequency on asthma and COPD (Supplementary Files S6 and S7).

### 2.7. Association Between Coffee Intake and COPD and Asthma

Coffee intake was not associated with COPD (OR = 1.18, 95% CI: 0.73–1.91, *p* = 0.506) and asthma (OR = 0.78, 95% CI: 0.55–1.11, *p* = 0.160) through the IVW method (Table 2). Other complementary approaches, such as MR Egger, weighted median, weighted mode, and simple mode, revealed similar conclusions. Cochran’s Q statistic indicated no significant heterogeneity among the IVs (*p* > 0.05) in the effect of coffee intake on asthma and COPD (Table 3). Similarly, the MR Egger intercept test for asthma and COPD showed no directional pleiotropy (*p* > 0.05). Scatter plot, funnel plot, and leave-one-out sensitivity plot are shown in Supplementary Figure S6.

## 3. Discussion

This study revealed that higher BMI, body fat percentage, and alcohol intake frequency were associated with greater risks of asthma and COPD, while fat-free mass specifically was associated with an elevated asthma risk, and body water mass and coffee consumption were not associated with either disease using the IVW approach. These results are consistent with several reports from MR studies that suggest that BMI is associated with increased risk of respiratory diseases [31,32,33]. Further analysis suggests that variants in *POC5* and *SLC39A8* may contribute to the observed associations between BMI and body fat percentage, and asthma and COPD. To investigate the functional implications of the rs13107325 (A391T) missense variant in *SLC39A8* and rs2307111 (H36R) in *POC5*, PolyPhen-2 analysis was performed. This analysis predicted that both substitutions were benign when the HumVar model was used.

Gene-based annotation evaluates the location of an SNP in relation to a gene, determining whether it is within or in proximity to the gene [34]. SNPs in coding regions, particularly nonsynonymous mutations, can change protein sequences, whereas those in regulatory regions, such as promoters and enhancers, can influence gene expression [35]. The majority of genomic loci found by GWAS for complex diseases are SNPs located in noncoding regions, particularly conserved introns, which, similar to exons, are subject to selective pressure and are potentially important to human health and adaptation [36]. About 50% of SNPs are found in noncoding regions, 25% result in missense mutations (coding SNPs), and the remaining 25% are silent mutations (synonymous SNPs), which do not alter the encoded amino acids [35]. A recent large-scale analysis indicated that approximately 32% (22.8 million) of 71 million surveyed missense variants were classified as likely pathogenic, while 57% (40.9 million) were considered likely benign [37]. This indicates that not all missense variants are pathogenic.

In the current study, some genetic variants that influenced the association of the exposure variables (BMI and body fat percentage) with asthma and COPD were single nucleotide variants, or missense variants in several genes, including the evolutionarily highly conserved gene *SLC39A8* (variant: rs13107325), specifically involving the C, A, and T alleles. *SLC39A8* encodes the ZIP8 cation transporter in all vertebrates [38], which is expressed in most mammalian tissues with higher expression in the lung and kidney in comparison to other tissues [39,40] (https://www.proteinatlas.org/ENSG00000138821-SLC39A8/tissue) (accessed on 18 May 2025). ZIP8 maintains an endogenous function that mediates the uptake of Mn^2+^, Zn^2+^, Fe^2+^, Se^4+^, and Co^2+^ into the cell [39]. Following the examination of all human zinc transporter transcripts (*SLC39A*_1–14_) and 10 exporters (*SLC30A*_1–10_) post-TNFα stimulation, only *SLC39A8* exhibited significant induction. This upregulation resulted in elevated intracellular zinc levels and conferred protection to primary human lung epithelial cells against apoptosis. However, *SLC39A8* siRNA resulted in increased cell mortality under inflammation onset [41]. As missense variants are alterations in the DNA sequence that result in a new amino acid in the protein, potentially modifying its function, this alteration may affect Zn^2+^ or other metal ion transport like Mn^2+^, thereby dysregulating immunological or inflammatory responses and increasing asthma or COPD susceptibility. Although the clinical significance of the gene variant is benign based on PolyPhen-2 analysis and may not likely affect protein function, experimental validation is warranted.

Existence of the *SLC39A8* genetic variant suggests potential genetic variability among individuals and may associate adiposity with asthma and COPD. A previous study identified a link between *SLC39A8* (rs13107325) and alcohol consumption, demonstrating a conserved role for *SLC39A8* in phenotypic responses to alcohol in model organisms such as *Caenorhabditis elegans* [42]. GWAS identified human variants of *SLC39A8* that have broad effects, exhibiting significant pleiotropy and affecting various clinical disorders across multiple organ systems like developmental and congenital disorders, immune system issues, cardiovascular problems, and conditions affecting the central nervous system, musculoskeletal system, eyes, and gastrointestinal tract [39]. Moreover, SLC39A8 deficiency (type II congenital disorder of glycosylation (CDG)) is a severe metabolic disorder caused by impaired manganese metabolism in humans, leading to multi-organ involvement and symptoms such as cranial synostoses with lacunar skull, cerebral and cerebellar atrophy, severe psychomotor disability, seizures, and vision and hearing impairment [43,44,45]. Key associated traits include manganese deficiency (leading to hypoglycosylation), congenital disabilities, mitochondrial dysfunction, lower HDL cholesterol, higher BMI, and increased risks for diseases such as coronary artery disease, stroke, schizophrenia, Parkinson’s, inflammatory bowel disease, myopia, scoliosis, lupus, and osteoarthritis [39]. As the *SLC39A8* variant is associated with the transport of zinc and manganese, potentially altering the susceptibility to metabolic and inflammatory disorders, this may influence immune cell function and the body’s response to inflammation.

**Figure 1:**
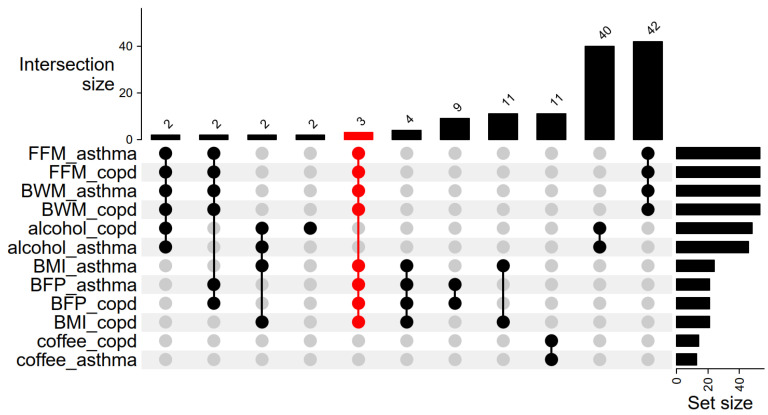
Overlapping SNPs with genes reveal intersections between body composition, beverage consumption, and lung diseases. BMI: body mass index; COPD: chronic obstructive pulmonary disease; FFM: fat-free mass; BFP: body fat percentage; BWM: body water mass. Red color indicates that a missense variant was identified in *POC5* and intronic variants in *NRXN3* and *FTO* among exposure variables: BMI, body fat percentage, body water mass, and fat-free mass.

Furthermore, across the body composition variables: BMI, body fat percentage, body water mass, and fat-free mass, a missense variant was identified in the overlapping genes *POC5*, and intronic variants in *NRXN3* and *FTO* (Figure 1). The *POC5* gene in humans encodes a protein that is universally expressed and predominantly found in centrioles where it interacts with centrin and inversin, making it crucial for assembling the distal portion of the centriole and for the elongation of centrioles [46]. POC5 contributes to various cellular processes, including cell polarity, division, and motility, and is an important component of the cytoskeleton, which is vital for cellular dynamics, while its localization in photoreceptors is vital for ciliary connectivity and retinal functionality [47,48]. Moreover, the *POC5* mutation is associated with the susceptibility to adolescent idiopathic scoliosis and has been shown to disrupt cell cycles, alter cilia length, and affect interactions among centrosome proteins [49,50].

GWAS studies revealed that the *POC5* variant is associated with pediatric-onset type 2 diabetes risk and the association between smoking and obesity susceptibility [51,52]. This suggests that obesity and metabolic dysregulation may link COPD and asthma via POC5, indicating its involvement in inflammation, immunological response, or metabolic control under both situations. As obesity is associated with asthma and exacerbations, a genetic mutation may explain their co-occurrence, supporting the hypothesis that obesity-related metabolic or inflammatory disorders may be associated with the acceleration of chronic lung diseases, especially in later-onset or severe instances. While additional SNPs linked to the association of BMI, body fat percentage, fat-free mass, and alcohol with asthma and COPD were identified to be missense variants in several genes (e.g., *ENPP2*, *ANAPC4*, *NCOR2*, *SH2B1*, *AATK*, *ADH1B*, *PPP2R2D*, and *ANAPC4*), as well as 3’UTR (e.g., *ZC3H4*, *ADCY9*, *RAB30*, *AGO2*, *HTT*, *ETFA*, *SNU13*, *FAIM2*, *TP53*, *NUFIP2*, and *KDM2A*) and 5’UTR variants (*GPR61*), these SNPs have not been extensively documented in CRDs, thus elucidating a potential direction for further functional research.

Obesity, which is considered a chronic systemic inflammatory state, is associated with airway inflammation and asthma by releasing pro-inflammatory cytokines from adipocytes, but direct evidence has not been demonstrated [53,54,55]. Moreover, to cater to the limitation posed by the failure of BMI to differentiate between adipose and muscle mass and, in some cases, overestimate or underestimate obesity, the association of body fat percentage with asthma and COPD was investigated. Similar to BMI, a positive association of body fat and COPD and asthma was demonstrated [56]. A longitudinal study conducted in Korea revealed that higher fat levels in adults increased airway hyperresponsiveness, a hallmark of asthma. This is possibly caused by inflammation, as adipose tissue in people with obesity has many macrophages, the majority of which are pro-inflammatory in asthma patients [57,58].

Fat-free mass influences energy balance. Ying et al. [59] reported that up to 85 diseases, including chronic lower respiratory diseases, were linked to genetically predicted fat-free mass using PheWAS analysis. Although there is no evidence of strong association between genetically predicted fat-free mass and COPD, the consistency of the three methods: IVW, MR Egger, and weighted mode, is indicative of the association between fat-free mass and the risk of asthma. This indicates a potential distinct pathophysiological relationship between fat-free mass and asthma.

Body water mass, readily assessed using bioimpedance, is a potential early indicator of preclinical disease, which has led to extensive research on its correlation with body fluid dysregulation and infectious disease outcomes in various populations [60]. While our study revealed no association of body water mass with COPD or asthma using the IVW method, two other MR approaches (MR Egger and weighted mode) indicated that increased body water mass is a risk factor for asthma. However, to our knowledge, MR studies examining the associations between body water mass and chronic respiratory diseases are limited. This indicates the need for further research on fluid intake monitoring as a critical factor that is associated with the prevention of both dehydration and overhydration, with implications for chronic respiratory conditions such as asthma.

Alcohol consumption influences the immune system and correlates with elevated immunoglobulin E (IgE) levels [18]. Additionally, it exhibits a U-shaped relationship with asthma risk, where moderate drinking is linked to the lowest risk [18]. In contrast to observational data, MR results indicate that modest alcohol consumption may not confer protective effects on obesity phenotypes, and excessive alcohol intake may be associated with higher incidence of obesity and an elevated risk of developing type 2 diabetes [61,62,63,64]. Alcohol intake frequency is associated with an increased risk of asthma and COPD. This suggests that alcohol intake frequency, in principle, is associated with an increased risk for asthma, COPD, and obesity, thereby elevating the likelihood of asthma development. Furthermore, alcohol use may contribute to the exacerbation of respiratory disease outcomes and increased morbidity, which also contribute to lengthier hospitalization periods. Although our results agree with some studies [18,19], the relationship between alcohol intake and asthma remains controversial as research findings are mixed, with some studies indicating no significant association [65,66]. The variability in these studies may be due to an emphasis on heavy alcohol consumption, confounding variables, and variations in experimental methodologies. Moreover, a recent study reported no association between alcohol intake frequency and COPD [4].

Coffee has been reported to offer potential health benefits due to the presence of bioactive compounds like caffeine, such as increased alertness, muscle strength, diuresis, and enhanced respiratory function [67]. In addition, theophylline, a metabolite of caffeine, is linked to dilating bronchi, stimulating respiratory centers, and anti-inflammation [67]. Due to its thermogenic effects, caffeine intake has been associated with weight reduction and lower risk of type 2 diabetes, cardiovascular diseases, and other diseases related to adiposity [68]. Although the current study did not indicate that coffee intake was associated with the risk of asthma and COPD, a few observational studies reported a protective effect of coffee intake on CRDs, such as asthma [69,70]. While some of these observational studies were adjusted for confounding factors like smoking and lifestyle, Lin et al. [69] noted that coffee intake was self-reported and did not consider additional sources of caffeine, including chocolate and other caffeinated beverages.

A major strength of this study was the use of MR analysis, which applies genetic variation as instrumental variables to deduce associations between variables, successfully mitigating bias from reverse causality and confounding, as genetic variants are established at conception and remain unaffected by disease status [71]. Furthermore, MR analysis precision was demonstrated by conducting sensitivity analysis to detect pleiotropy, such as MR Egger regression and MRPRESSO, and estimated SNPs that are associated with the exposures. To mitigate needless bias in exposures and outcomes, European populations from different countries were evaluated. However, this perceived strength also constitutes a major drawback due to limited generalizability to other populations. While MR demonstrates a strong association between BMI, body fat percentage, and fat-free mass with CRDs like asthma and COPD, the magnitude of this link does not elucidate the potential effects of clinical or public health interventions. Additionally, the absence of age- or sex-stratified analysis to categorize significant disparities in population characteristics of the exposures and outcomes may further limit the interpretation of the results and validity of associations within specific demographics. Lastly, although SNPs serve as instrumental variables in this study, most SNPs do not have direct biological consequences. When only SNPs are analyzed or utilized, it is important to recognize that other variants which have stronger effects may have been missed. Hence, there is a need for cautious interpretation of the results from the SNP annotation pending experimental validation.

## 4. Methods

The GWAS summary-level data that was used in this study was obtained from the IEU OpenGWAS project (https://gwas.mrcieu.ac.uk/) (accessed on 24 April 2025), developed by the MRC Integrative Epidemiology Unit (IEU) at the University of Bristol. The platform features a manually curated collection of complete GWAS summary datasets and compiles and analyzes data from the UK Biobank and the FinnGen biobank. A token was obtained from the OpenGWAS API for programmatic access to the IEU OpenGWAS database.

### 4.1. Data Sources

The GWAS summary-level data of body composition metrics and dietary factors serves as the exposure used in the study. The exposure was extracted from UK Biobank by the IEU OpenGWAS project and included body fat percentage, BMI, fat-free mass, and body water mass, in addition to dietary factors such as alcohol intake frequency and coffee intake. The GWAS summary-level data of the outcomes, asthma and COPD, were extracted from the FinnGen biobank by the IEU OpenGWAS project. No proxy SNPs were used to identify SNPs from the outcome as the FinnGen biobank dataset for asthma and COPD has a sufficient number of SNPs (Table 1). The population from the two studies is of European descent, with no overlapping populations between exposures and outcomes [30]. As the data used was public, anonymized, and de-identified, no ethical permission was requested, and the study followed the STROBE-MR guidelines [29].

### 4.2. Selection of Instrumental Variables

**Figure 2:**
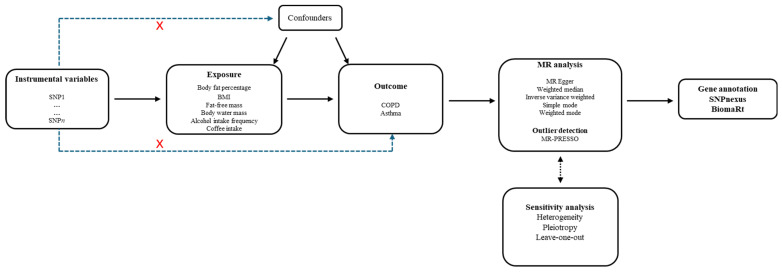
Schematic overview of the Mendelian randomization analysis. MR: Mendelian randomization; BMI: body mass index; COPD: chronic obstructive pulmonary disease; SNP: single nucleotide polymorphism; PRESSO: Pleiotropy Residual Sum and Outlier. X: strong association with the exposure of interest, association with the outcome only via the exposure (indicating no horizontal pleiotropy), and lack of association with any confounder.

First, SNPs significantly associated with exposures (body fat percentage, BMI, fat-free mass, body water mass, alcohol intake frequency, and coffee intake) were screened at the genome-wide significance level of *p* < 5 × 10^−8^, with a clumping window > 10,000 kb, and a linkage disequilibrium level (r2 < 0.001) [27], following the steps shown in Figure 2. F-statistics were used to assess the extent of weak instrument bias of the IVs [4]. To reduce the bias caused by weak IVs, the working variables with F > 10 were retained for alcohol and coffee intake, while the SNPs with F > 100 were retained for body fat percentage, BMI, fat-free mass, and body water mass datasets with more SNPs. F-values < 10 were excluded from the analysis as they indicate weak instrument bias [19]. Additional queries were conducted utilizing SNPnexus to ascertain SNPs linked to established confounding variables, and SNP-gene annotation was obtained from the Ensembl database via the biomaRt package [72,73,74]. Moreover, possible impact of an amino acid substitution on the structure and function of some proteins were determined using PolyPhen-2 [75]. SNPs with no specified or mapped association were deleted, while only those with significant associations such as missense variant, intron variant, and 3 and 5’ UTR variant were retained.

### 4.3. Statistical Analysis

MR Egger, weighted median, inverse variance weighted (IVW), simple mode, and weighted mode were used to examine the causal association between exposure and outcome. Each IV was aligned with the same effect alleles prior to MR analysis. The IVW model exhibits the highest capacity for detecting causality in the two-sample MR analysis, requiring either all variants to be valid instruments or a balanced horizontal pleiotropy, and was selected as the primary method for the MR analysis [76]. The weighted median and MR Egger regression approaches were used to evaluate the robustness of the results [27]. Moreover, the conclusion of the study was strengthened if the results of the five MR models were consistent. The variability of the IVW model was evaluated using Cochran’s Q test, with *p* < 0.05 signifying heterogeneity. Nonetheless, the presence of heterogeneity does not imply that the IVW model is inherently flawed [19]. The favorable assessment of the outcomes necessitates that, at minimum, the IVW approach yields statistically significant (*p* < 0.05) results, and the directions of the beta values derived from the weighted median and MR Egger analyses are congruent [4,32]. Furthermore, a strong horizontal pleiotropy in the MR analysis is indicated by a statistically significant MR Egger intercept. A leave-one-out analysis examined whether any one SNP caused the overall effect, whereas MR-PRESSO eliminated outliers and the data were reanalyzed. All analyses were performed using the TwoSampleMR package in R software (version 4.4.2).

### 4.4. Enrichment Analysis

SNPs in the final dataset that were mapped to genic regions were used as input to Gene Ontology (GO) and KEGG pathway enrichment analysis. Enrichment analyses were conducted using clusterProfiler v4.14.4 [77] and the genome-wide annotation for the human genome that is available through Bioconductor Annotation Data Packages (Org.Hs.eg.db). The Benjamini–Hochberg method was used to correct *p*-values for multiple comparisons. Because the SNP results for FFM-COPD, FFM-Asthma, BWM-COPD, and BWM asthma were extremely similar, the intersection of all genes associated with these groups were used for enrichment analysis (52 out of 54 total genes). Genes belonging to overlapping groups were visualized using ComplexHeatmap v2.22 [78].

## 5. Conclusions

The identification of missense variants, rs13107325 and rs2307111 in *SLC39A8* and *POC5,* respectively, suggests that these genes may have some associations or roles in the development of CRDs, including asthma and COPD. While the precise roles and functions remain to be elucidated, exploring the potential links between these genes, immune modulation, and inflammation may contribute to the identification of biomarkers that are associated with susceptibility to and progression of chronic lung disease, particularly in individuals with metabolic dysregulation such as obesity. Moreover, genetic screening for *SLC39A8* and *POC5* may provide insight into patient stratification and inform the development of targeted interventions aimed at immunological and inflammatory pathways in lung disease.

## Figures and Tables

**Table 1:** Overview of the exposures and outcome GWAS summary-level data used in the MR analyses for the exposures.

GWAS ID	Year	Trait	Consortium	Sample Size	SNPs
ukb-b-19953	2018	Body mass index	MRC-IEU	461,460	9,851,867
ukb-b-8909	2018	Body fat percentage	MRC-IEU	454,633	9,851,867
ukb-b-13354	2018	Whole-body fat-free mass	MRC-IEU	454,850	9,851,867
ukb-b-14540	2018	Whole-body water mass	MRC-IEU	454,888	9,851,867
ukb-b-5237	2018	Coffee intake	MRC-IEU	428,860	9,851,867
ukb-b-5779	2018	Alcohol intake frequency	MRC-IEU	462,346	9,851,867
finn-b-J10_ASTHMA_MAIN_EXMORE	2021	Asthma (only as main-diagnosis), excluding more control		Cases = 17,438 Controls = 131,051	16,380,048
finn-b-K11_CD_NOUC	2021	Crohn disease (strict definition, all UC cases excluded)		Cases = 657 Controls = 210,300	16,380,454

**Table 2:** MR analysis results to estimate causal impacts of adiposity, body composition metrics, and beverage consumption on chronic respiratory disease risk.

Exposures	ID	Outcomes	SNPs	MR Analysis				
				Method	b	se	*p* Value	OR (95% CI)
BFP	ukb-b-8909	COPD	36	MR Egger	0.37	0.71	0.609	1.44 (0.36, 5.76)
				weighted median	0.40	0.24	0.094	1.49 (0.94, 2.37)
				Inverse variance weighted	0.54	0.17	0.002	1.72 (1.23, 2.41)
				simple mode	0.80	0.51	0.125	2.22 (0.82, 6.0)
				weighted mode	0.29	0.40	0.478	1.34 (0.61, 2.95)
BFP	ukb-b-8909	Asthma	36	MR Egger	0.66	0.56	0.248	1.93 (0.65, 5.78)
				weighted median	0.54	0.17	0.002	1.72 (1.23, 2.41)
				Inverse variance weighted	0.47	0.14	0.001	1.60 (1.23, 2.09)
				simple mode	0.11	0.43	0.807	1.11 (0.48, 2.56)
				weighted mode	0.84	0.31	0.011	2.32 (1.25, 4.29)
BMI	ukb-b-19953	COPD	38	MR Egger	0.17	0.31	0.585	1.18 (0.65, 2.16)
				weighted median	0.28	0.17	0.109	1.32 (0.94, 1.85)
				Inverse variance weighted	0.44	0.12	0.000	1.56 (1.23, 1.98)
				simple mode	0.19	0.37	0.621	1.20 (0.58, 2.49)
				weighted mode	0.10	0.22	0.643	1.11 (0.72, 1.70)
BMI	ukb-b-19953	Asthma	42	MR Egger	0.41	0.24	0.095	1.50 (0.94, 2.40)
				weighted median	0.52	0.11	0.000	1.68 (1.35, 2.10)
				Inverse variance weighted	0.43	0.09	0.000	1.54 (1.29, 1.84)
				simple mode	0.60	0.27	0.033	1.83 (1.07, 3.11)
				weighted mode	0.50	0.15	0.002	1.65 (1.23, 2.19)
FFM	ukb-b-13354	COPD	98	MR Egger	0.33	0.39	0.398	1.39 (0.65, 2.96)
				weighted median	0.25	0.17	0.129	1.29 (0.93, 1.78)
				Inverse variance weighted	0.24	0.13	0.073	1.27 (0.98, 1.64)
				simple mode	0.48	0.43	0.269	1.62 (0.69, 3.80)
				weighted mode	0.41	0.36	0.255	1.51 (0.75, 3.04)
FFM	ukb-b-13354	Asthma	99	MR Egger	0.59	0.26	0.023	1.81 (1.10, 2.98)
				weighted median	0.12	0.11	0.273	1.13 (0.91, 1.40)
				Inverse variance weighted	0.19	0.09	0.032	1.21 (1.02, 1.44)
				simple mode	−0.32	0.35	0.365	0.73 (0.37, 1.44)
				weighted mode	0.61	0.29	0.037	1.84 (1.05, 3.23)
BWM	ukb-b-14540	COPD	97	MR Egger	0.33	0.39	0.393	1.40 (0.65, 2.99)
				weighted median	0.22	0.17	0.195	1.25 (0.89, 1.75)
				Inverse variance weighted	0.24	0.13	0.071	1.27 (0.98, 1.65)
				simple mode	0.44	0.43	0.311	1.55 (0.67, 3.58)
				weighted mode	0.36	0.37	0.328	1.44 (0.70, 2.97)
BWM	ukb-b-14540	Asthma	98	MR Egger	0.71	0.25	0.006	2.04 (1.24, 3.34)
				weighted median	0.11	0.12	0.370	1.11 (0.88, 1.40)
				Inverse variance weighted	0.16	0.09	0.068	1.18 (0.99, 1.40)
				simple mode	−0.32	0.31	0.317	0.73 (0.40, 1.35)
				weighted mode	0.57	0.28	0.043	1.77 (1.03, 3.04)
Alcohol	ukb-b-5779	COPD	87	MR Egger	0.47	0.35	0.178	1.60 (0.81, 3.14)
				weighted median	0.18	0.16	0.260	1.20 (0.88, 1.64)
				Inverse variance weighted	0.30	0.11	0.009	1.34 (1.08, 1.68)
				simple mode	−0.30	0.42	0.478	0.74 (0.33, 1.69)
				weighted mode	−0.27	0.36	0.467	0.77 (0.38, 1.56)
Alcohol	ukb-b-5779	Asthma	86	MR Egger	0.47	0.26	0.073	1.60 (0.96, 2.65)
				weighted median	0.22	0.11	0.040	1.25 (1.01, 1.54)
				Inverse variance weighted	0.17	0.08	0.039	1.19 (1.01, 1.40)
				simple mode	0.22	0.27	0.422	1.24 (0.74, 2.09)
				weighted mode	0.29	0.18	0.113	1.33 (0.94, 1.90)
Coffee	ukb-b-5237	COPD	36	MR Egger	−0.14	0.48	0.778	0.87 (0.34, 2.25)
				weighted median	0.00	0.34	0.996	1.00 (0.51, 1.97)
				Inverse variance weighted	0.16	0.25	0.506	1.18 (0.73, 1.91)
				simple mode	−0.24	0.68	0.724	0.79 (0.21, 2.95)
				weighted mode	−0.03	0.35	0.941	0.98 (0.50, 1.92)
Coffee	ukb-b-5237	Asthma	36	MR Egger	−0.23	0.35	0.525	0.80 (0.40, 1.59)
				weighted median	−0.19	0.23	0.411	0.83 (0.53, 1.30)
				Inverse variance weighted	−0.25	0.18	0.160	0.78 (0.55, 1.11)
				simple mode	−0.34	0.47	0.473	0.71 (0.28, 1.80)
				weighted mode	−0.33	0.23	0.164	0.72 (0.46, 1.13)

BFP, body fat percentage; BMI, body mass index; FFM, fat-free mass; BWM, body water mass; CI, confidence intervals; MR, Mendelian randomization; OR, odds ratio; COPD, chronic obstructive pulmonary disease; SNPs, single nucleotide polymorphisms; b, intercept; se, standard error.

**Table 3:** Sensitivity analyses of adiposity, body composition metrics, and beverage consumption on chronic respiratory disease risk.

Exposures	ID	Outcomes	SNPs	Sensitivity Analysis				
				Heterogeneity Test		MR Egger Pleiotropy Test		
				Cochrane Q	*p*-Value	b	se	*p*-Value
BFP	ukb-b-8909	COPD	36	38.96	0.296	0.00	0.02	0.799
		Asthma	36	55.04	0.017	−0.00	0.01	0.732
BMI	ukb-b-19953	COPD	38	44.76	0.178	0.01	0.01	0.334
		Asthma	42	67.97	0.005	0.00	0.01	0.919
FFM	ukb-b-13354	COPD	98	152.95	0.000	−0.00	0.01	0.804
		Asthma	99	156.01	0.000	−0.01	0.01	0.097
BWM	ukb-b-14540	COPD	97	153.20	0.000	−0.00	0.01	0.799
		Asthma	98	154.31	0.000	−0.01	0.01	0.022
Alcohol	ukb-b-5779	COPD	87	116.93	0.015	−0.00	0.01	0.597
		Asthma	86	131.24	0.001	−0.01	0.01	0.228
Coffee	ukb-b-5237	COPD	36	40.04	0.256	0.01	0.01	0.471
		Asthma	36	46.90	0.086	0.00	0.01	0.933

BFP, body fat percentage; BMI, body mass index; FFM, fat-free mass; BWM, body water mass; MR, Mendelian randomization; COPD, chronic obstructive pulmonary disease; SNPs, single nucleotide polymorphisms; b, intercept; se, standard error.

## Data Availability

GWAS summary statistics are available from the IEU OpenGWAS project (https://gwas.mrcieu.ac.uk/).

## Supplementary Materials

The following supporting information can be downloaded at: https://www.mdpi.com/article/10.3390/ijms26167799/s1.

## Abbreviations

MRC, medical research council; IEU, integrative epidemiology unit; GWAS, genome-wide association study; COPD, chronic obstructive pulmonary disease; SNP, single nucleotide polymorphism; MR, Mendelian randomization; BMI, body mass index; IVW, inverse variance weighted; CRD, chronic respiratory disease; MTMR11, myotubularin related protein 11; SLC39A8, solute carrier family 39 member 8; POC5, Protein of centriole 5; WSCD2, WSC domain-containing protein 2; SH2B1, Src Homology 2 (SH2) domain-containing transforming protein B adaptor protein 1; FAIM2, Fas Apoptotic Inhibitory Molecule 2; UTR, untranslated region; ANAPC4, Anaphase Promoting Complex Subunit 4; ADCY9, Adenylate Cyclase 9; GPR61, G Protein-Coupled Receptor 61; TAOK2, TAO Kinase 2; ENPP2, Ectonucleotide Pyrophosphatase/Phosphodiesterase 2; NCOR2, Nuclear Receptor Corepressor 2; AATK, Apoptosis Associated Tyrosine Kinase; ZC3H4, Zinc Finger CCCH-Type Containing 4; SNU13, Small nuclear ribonucleoprotein 13; TP53, Tumor Protein p53; KDM2A, Lysine Demethylase 2A; MR-PRESSO, Mendelian randomization pleiotropy residual sum and outlier; ADH1B, Alcohol Dehydrogenase 1B (Class I); PPP2R2D, Protein Phosphatase 2 Regulatory Subunit B, Delta; RAB30, Member RAS Oncogene Family; AGO2, Argonaute RISC Catalytic Component 2; HTT, Huntingtin; ETFA, electron transfer flavoprotein alpha subunit; NUFIP2, nuclear fragile X mental retardation-interacting protein 2; ZIP8, zinc transporter zinc-and iron-related protein 8; FTO, fat mass and obesity-associated gene; NRXN3, Neurexin 3.
