# Supplementary material for: Exploring the Potential Roles of SLC39A8 and POC5 Missense Variants in the Association Between Body Composition, Beverage Consumption, and Chronic Lung Diseases: A Two-Sample Mendelian Randomization Study
Source: Int J Mol Sci. 2025 Aug 12;26(16):7799. doi: 10.3390/ijms26167799 (PMC12386338; doi:10.3390/ijms26167799)
Supplement: Supplementary file 1 [file ijms-26-07799-s001.zip › Supplementary Figures S1-S6.pdf]

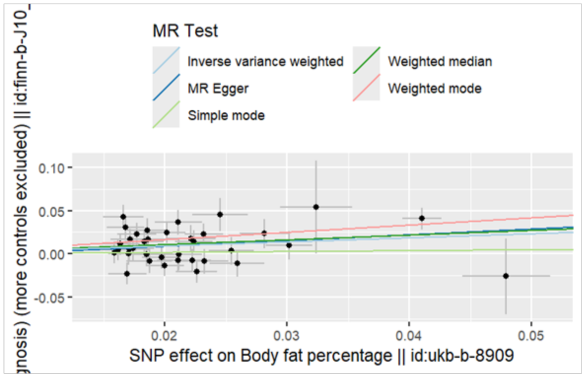

(a) Scatter plot showing the causal effect of body fat percentage on Asthma.

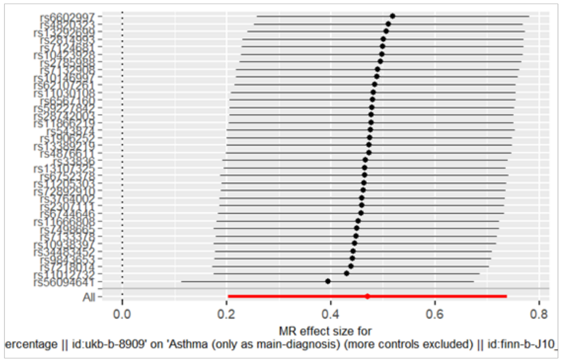

(b) Leave-one-out analysis of body fat percentage on asthma.

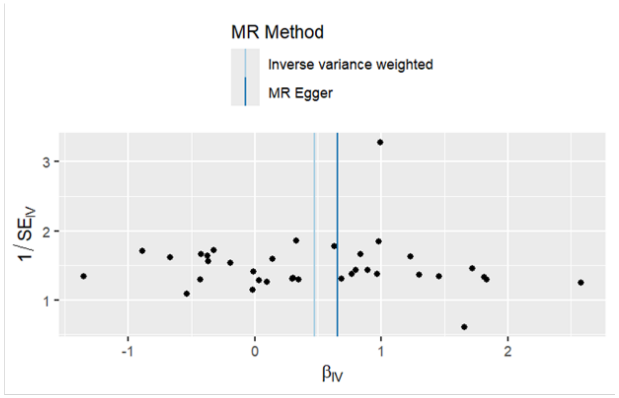

(c) Funnel plot of SNPs associated with body fat percentage on the risk of asthma.

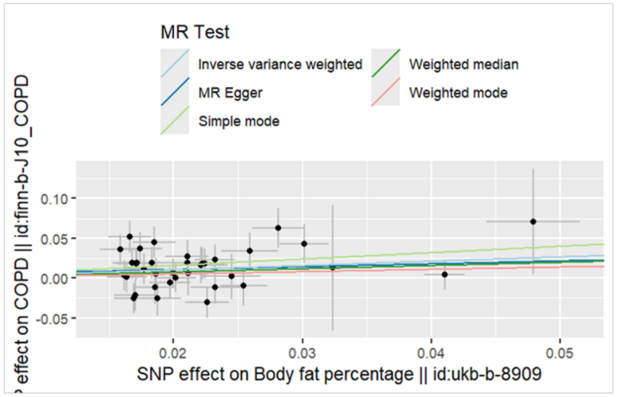

(d) Scatter plot showing the causal effect of body fat percentage on COPD.

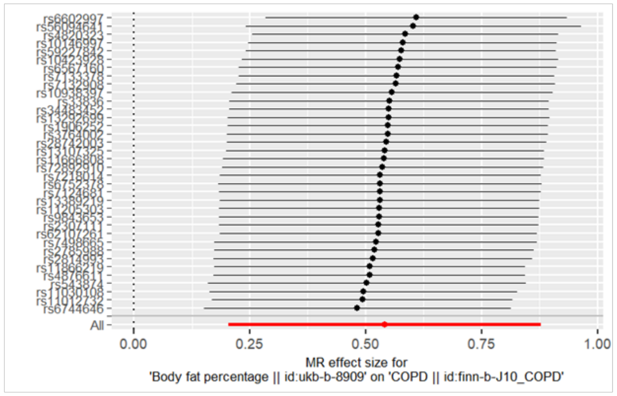

(e) Leave-one-out analysis of body fat percentage on COPD.

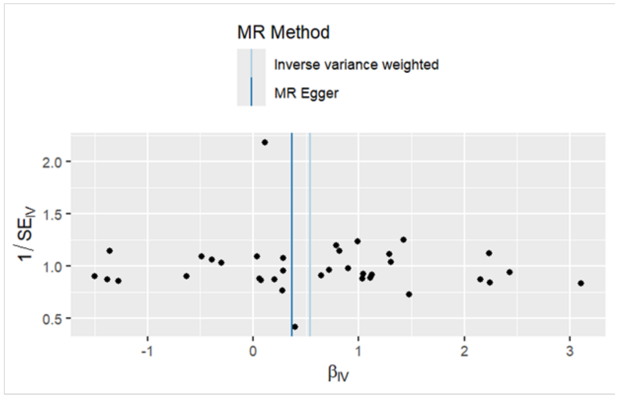

(f) Funnel plot of SNPs associated with body fat percentage on the risk of COPD.

Figure S1. MR analysis and sensitivity plots of body fat percentage on Asthma (a-c) and COPD (d-f).

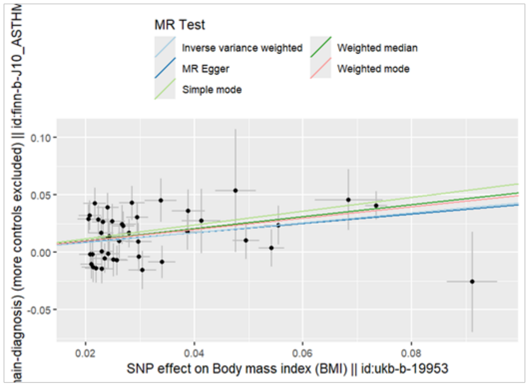

(a) Scatter plot showing the causal effect of BMI on Asthma.

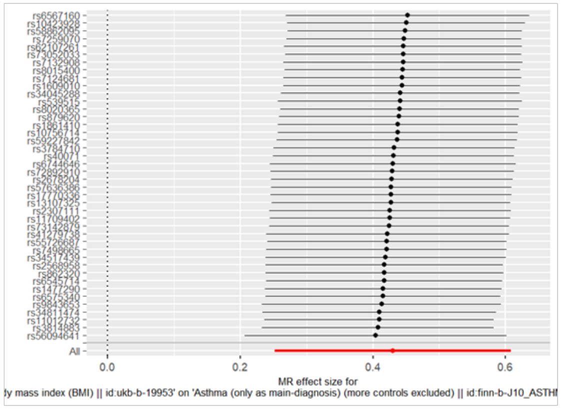

(b) Leave-one-out analysis of BMI on asthma.

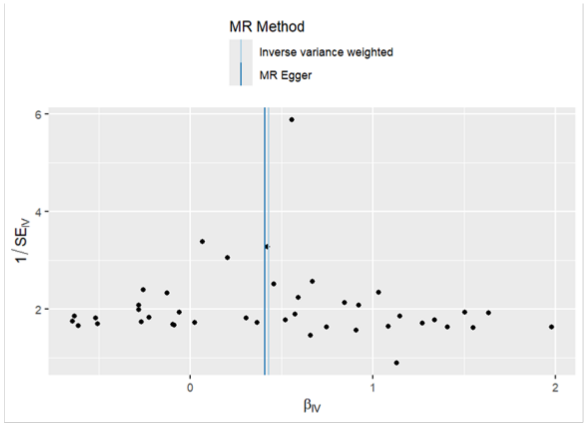

(c) Funnel plot of SNPs associated with BMI on the risk of asthma.

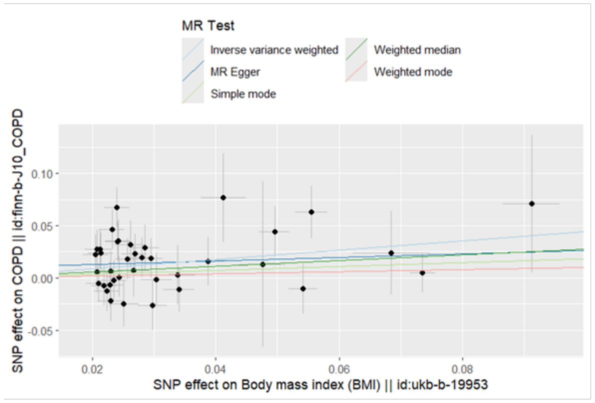

(d) Scatter plot showing the causal effect of BMI on COPD.

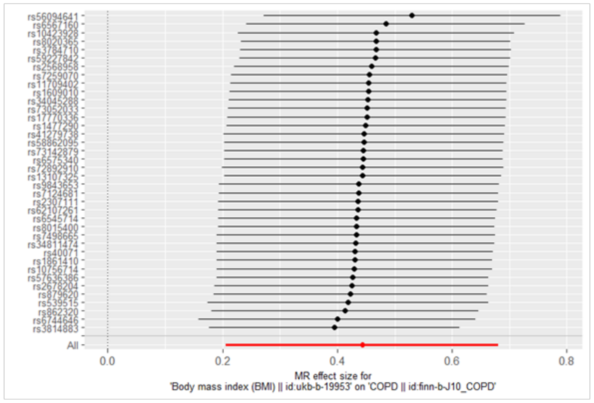

(e) Leave-one-out analysis of BMI on COPD.

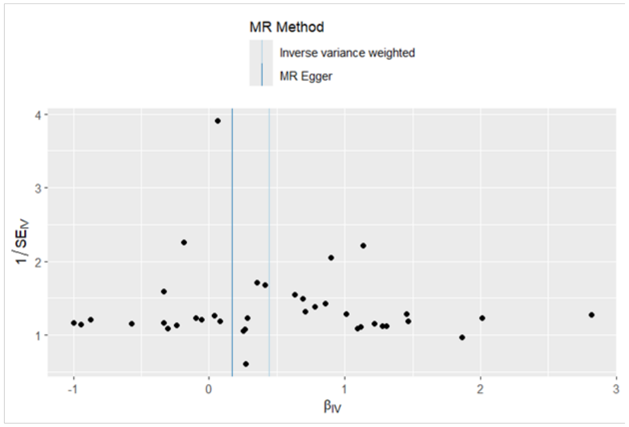

(f) Funnel plot of SNPs associated with BMI on the risk of COPD.

Figure S2. MR analysis and sensitivity plots of BMI on Asthma (a-c) and COPD (d-f).

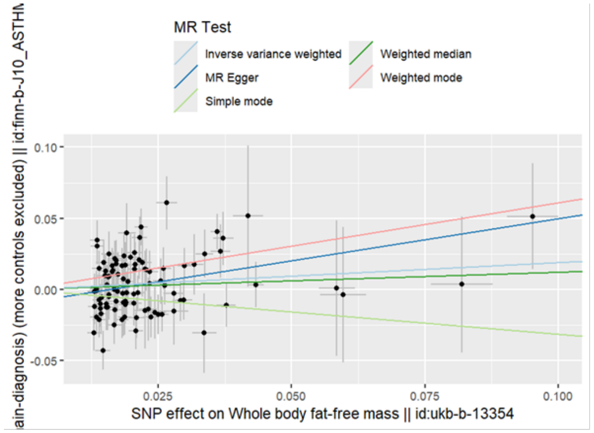

(a) Scatter plot showing the causal effect of fat-free mass on Asthma.

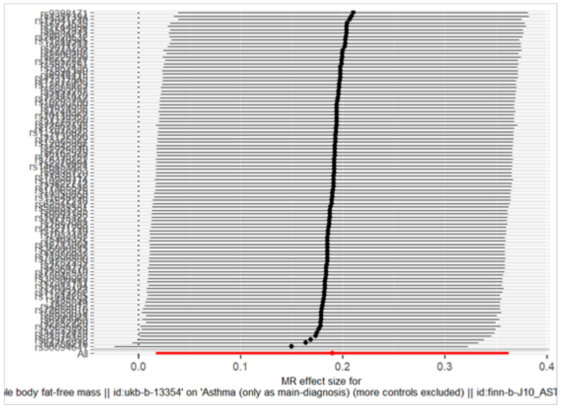

(b) Leave-one-out analysis of fat-free mass on asthma.

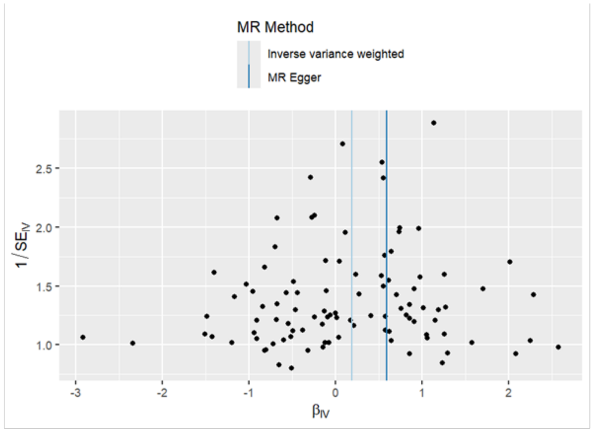

(c) Funnel plot of SNPs associated with fat-free mass on the risk of asthma.

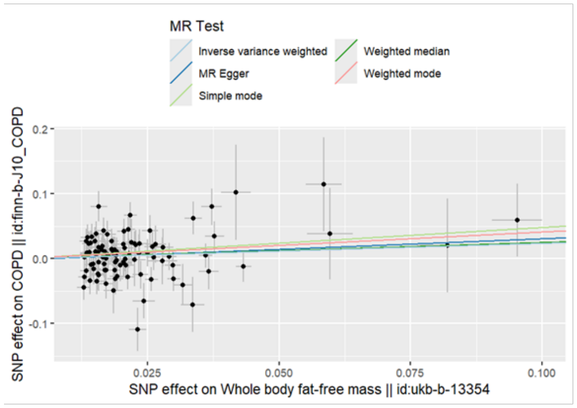

(d) Scatter plot showing the causal effect of fat-free mass on COPD.

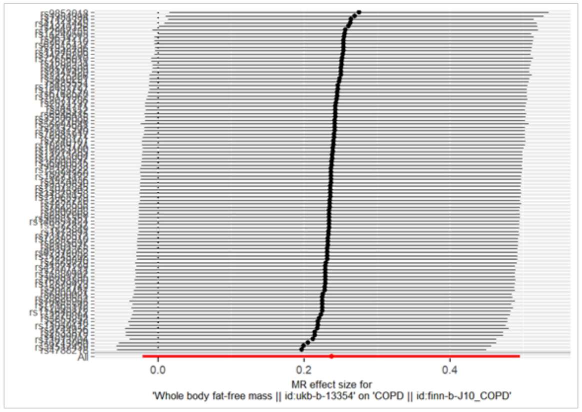

(e) Leave-one-out analysis of fat-free mass on COPD.

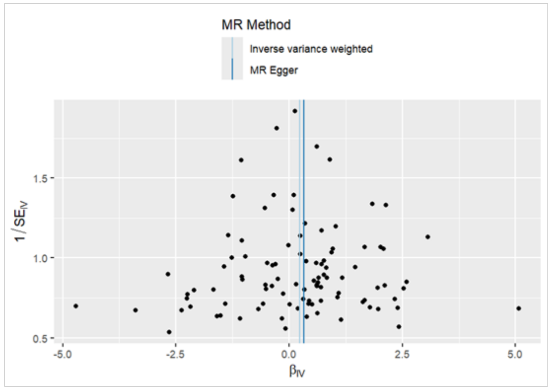

(f) Funnel plot of SNPs associated with fat-free mass on the risk of COPD.

Figure S3. MR analysis and sensitivity plots of fat-free mass on Asthma (a-c) and COPD (d-f).

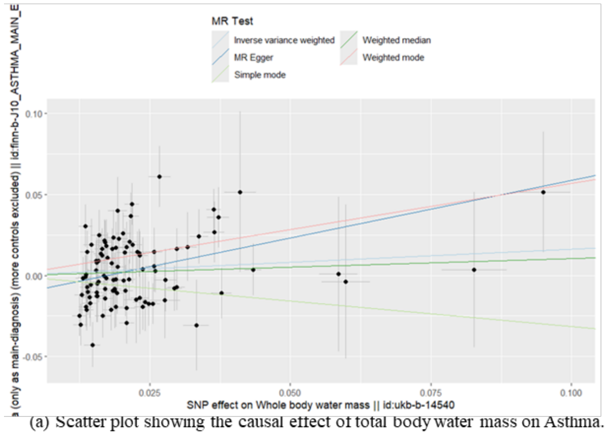

(a) Scatter plot showing the causal effect of total body water mass on Asthma.

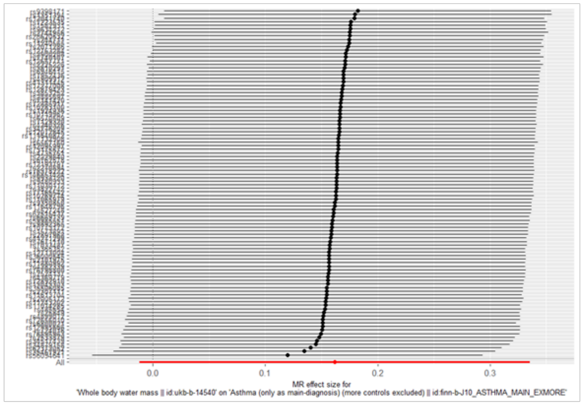

(b) Leave-one-out analysis of total body water mass on asthma.

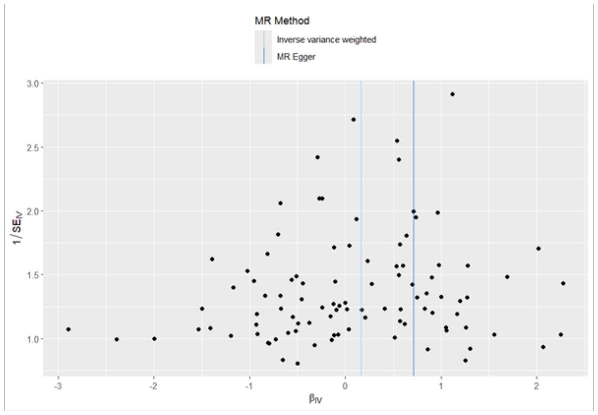

(c) Funnel plot of SNPs associated with total body water mass on the risk of asthma.

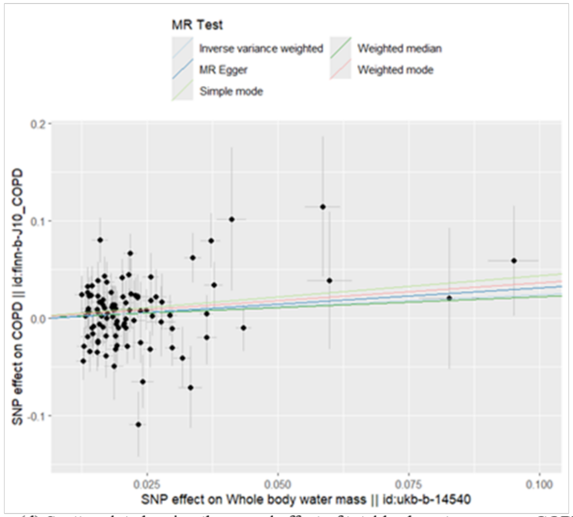

(d) Scatter plot showing the causal effect of total body water mass on COPD.

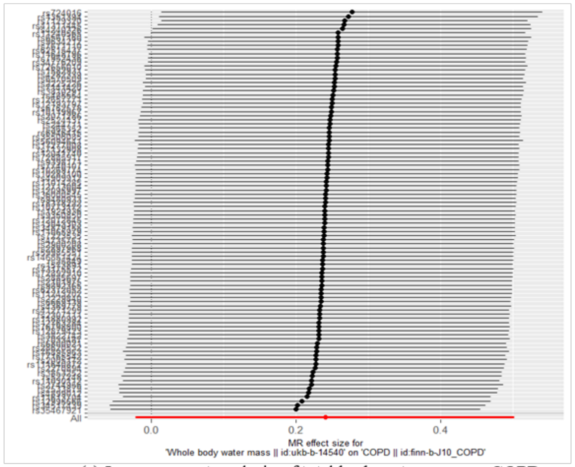

(e) Leave-one-out analysis of total body water mass on COPD.

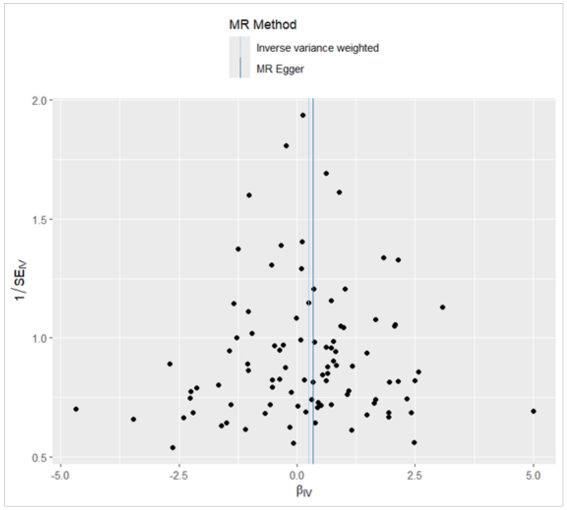

(f) Funnel plot of SNPs associated with total body water mass on the risk of COPD.

Figure S4. MR analysis and sensitivity plots of total body water mass on asthma (a-c) and COPD (d-f).

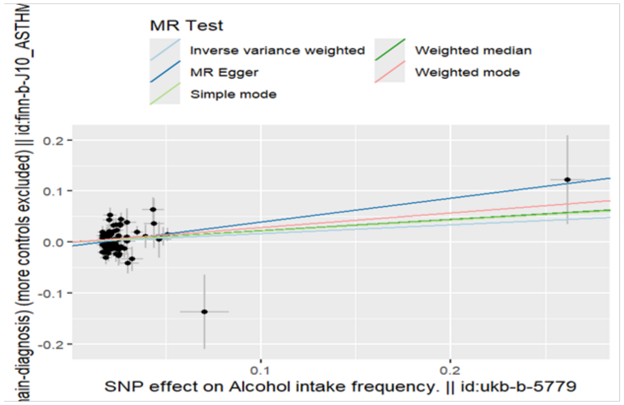

(a) Scatter plot showing the causal effect of alcohol intake frequency on Asthma.

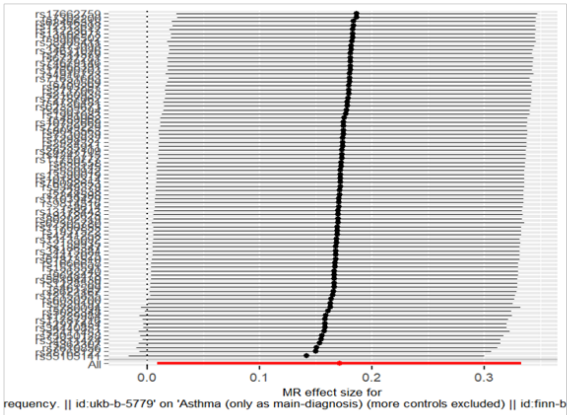

(b) Leave-one-out analysis of alcohol intake frequency on asthma.

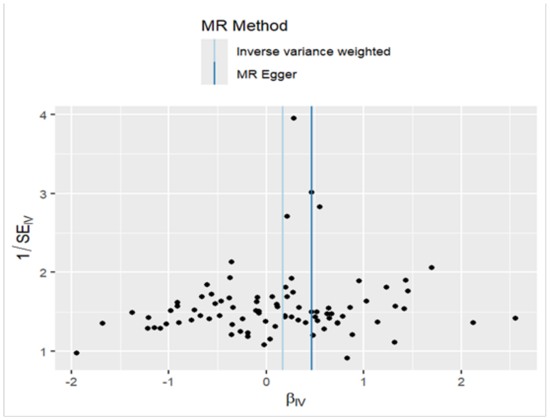

(c) Funnel plot of SNPs associated with alcohol intake frequency on the risk of asthma.

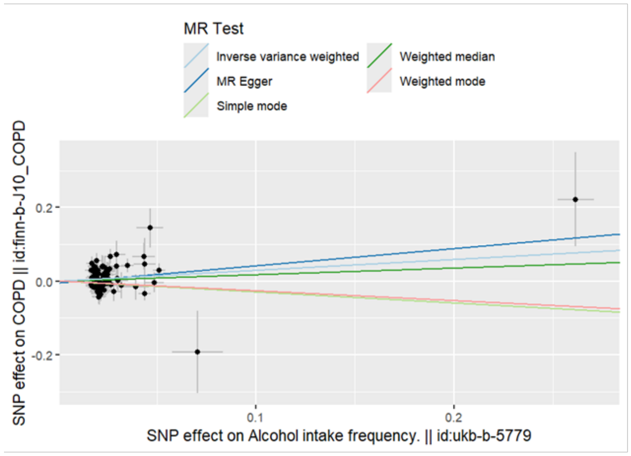

(d) Scatter plot showing the causal effect of alcohol intake frequency on COPD.

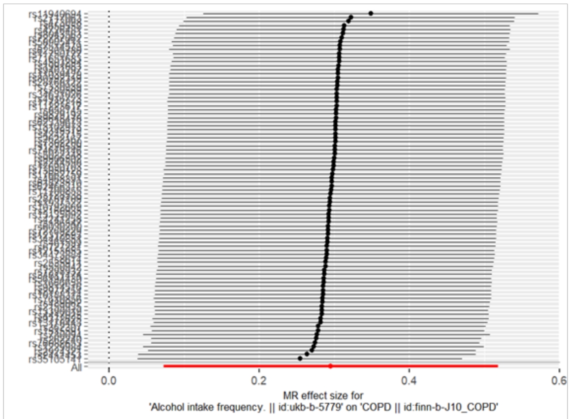

(e) Leave-one-out analysis of alcohol intake frequency on COPD.

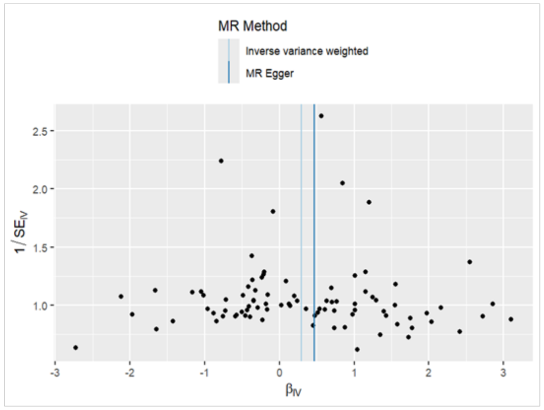

(f) Funnel plot of SNPs associated with alcohol intake frequency on the risk of COPD.

Figure S5. MR analysis and sensitivity plots of alcohol intake frequency on asthma (a-c) and COPD (d-f).

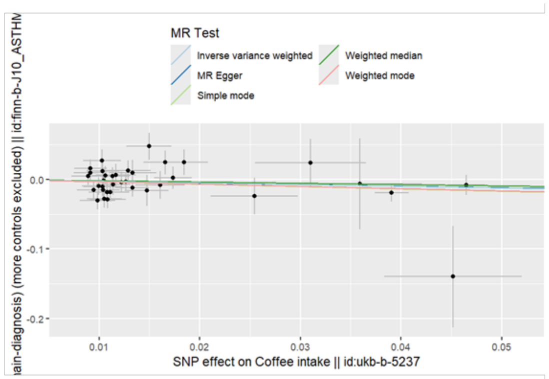

(a) Scatter plot showing the causal effect of coffee intake on Asthma.

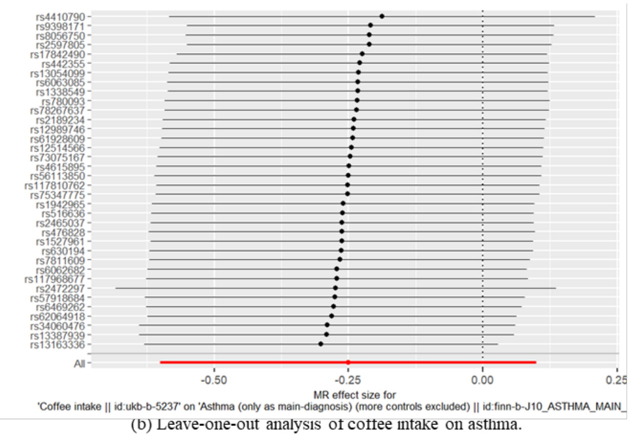

(b) Leave-one-out analysis of coffee intake on asthma.

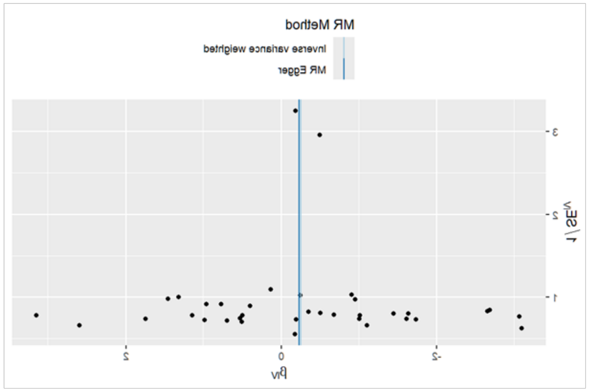

(c) Funnel plot of SNPs associated with coffee intake on the risk of asthma.

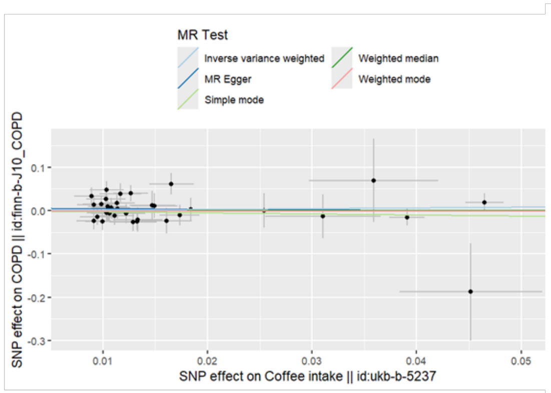

(d) Scatter plot showing the causal effect of coffee intake on COPD.

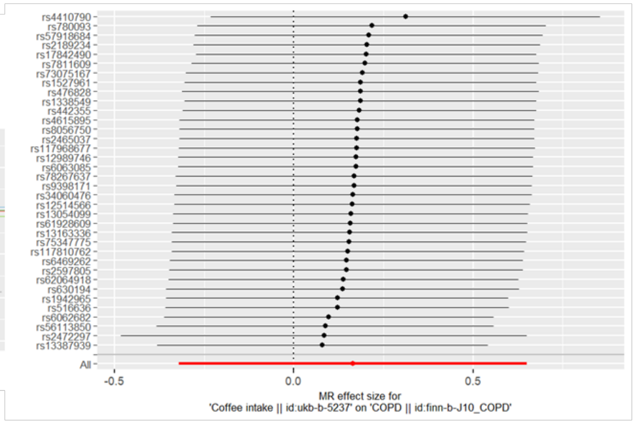

(e) Leave-one-out analysis of coffee intake on COPD.

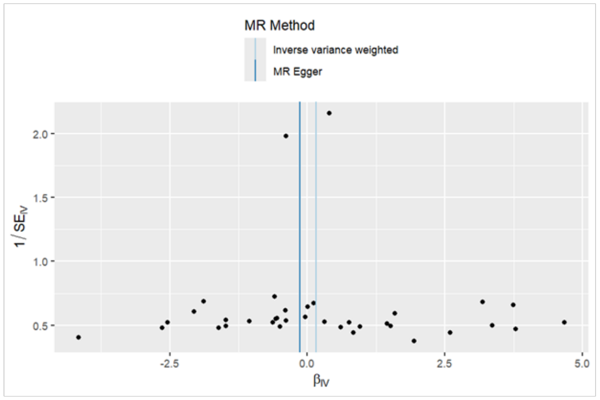

(f) Funnel plot of SNPs associated with coffee intake on the risk of COPD.

Figure S6. MR analysis and sensitivity plots of coffee intake on asthma (a-c) and COPD (d-f).
